# Attosecond electron microscopy and diffraction

**DOI:** 10.1126/sciadv.adp5805

**Published:** 2024-08-21

**Authors:** Dandan Hui, Husain Alqattan, Mohamed Sennary, Nikolay V. Golubev, Mohammed Th. Hassan

**Affiliations:** Department of Physics, University of Arizona, Tucson, AZ 85721, USA.

## Abstract

Advances in attosecond spectroscopy have enabled tracing and controlling the electron motion dynamics in matter, although they have yielded insufficient information about the electron dynamic in the space domain. Hence, ultrafast electron and x-ray imaging tools have been developed to image the ultrafast dynamics of matter in real time and space. The cutting-edge temporal resolution of these imaging tools is on the order of a few tens to a hundred femtoseconds, limiting imaging to the atomic dynamics and leaving electron motion imaging out of reach. Here, we obtained the attosecond temporal resolution in the transmission electron microscope, which we coined “attomicroscopy.” We demonstrated this resolution by the attosecond diffraction measurements of the field-driven electron dynamics in graphene. This attosecond imaging tool would provide more insights into electron motion and directly connect it to the structural dynamics of matter in real-time and space domains, opening the door for long-anticipated real-life attosecond science applications in quantum physics, chemistry, and biology.

## Teaser

Attosecond electron microscopy is demonstrated and used to image the graphene electron motion in action.

## INTRODUCTION

The advancement in ultrafast science opened a window to see the ultrafast dynamics of matter span from the material phase transitions, molecular, atomic, and electronic motions in real time (*1*–*8*). This capability has been extended to include the space dimensions by developing ultrafast x-ray and electron imaging tools (*9*–*11*). Ultrafast electron microscopy (UEM) is one of these crucial tools to envisage the evolution of the matter dynamics in the four dimensions of space and time (*12*–*16*). The basic idea of UEM depends on generating ultrafast bursts of electrons inside the microscope by laser pulses to image the matter’s dynamics triggered by another pump laser pulse via time-resolved measurements (*17*, *18*). Hence, the imaging temporal resolution is defined by the temporal duration of the electron pulses, not the mechanical shatter of the microscope detector.

At the beginning of the century, the first demonstration of UEM with nanosecond resolution was reported to image the laser-induced melting of nickel metal and the morphology change of cobalt bulk (*17*). After this pioneer work, many research efforts were focused on enhancing the temporal resolution of UEM. In 2008, the temporal resolution of UEM was enhanced to a subpicosecond time scale, extending the capability of UEM to image faster laser-induced molecular and atomic motions (*18*). Although further enhancement of the UEM temporal resolution was technically challenging due to the crucial space charge effect because of traveling the ultrafast electron wave packet inside the microscope from the source to the sample. Hence, many research efforts focused on compressing the electron pulses (*19*–*37*) and developing high-bright photoemission electron sources, such as laser-driven Schottky field emission and cold field-emission guns using the apex of the nanoemitter to generate a few hundred femtoseconds high-bright electron pulses (*38*, *39*). Then, the optical-gating approach has been demonstrated to enhance the temporal resolution of UEM by an order of magnitude to a few tens of femtoseconds (*31*, *40*). This technique is based on controlling the temporal profile and gating the electrons inside the microscope by ultrashort laser pulses in the time domain. This enhancement opened the door to image the collective electron dynamics, such as surface plasmon electron dynamics (*41*). However, imaging the electron motion requires an enhancement of the UEM temporal resolution to the subfemtosecond time scale. A train of attosecond electron pulses has been demonstrated in UEM using the gating approach by continuous and pulsed laser beams by other groups (*32*, *36*, *37*, *42*, *43*). This train of attosecond electron pulses contains (*n*) number of electron pulses. Hence, using this pulse train of electrons in time-resolved ultrafast imaging measurement, the dynamical process will be probed (*n*) times at every single triggering (pumping) event. Accordingly, the recorded snapshots form a video of the average dynamics (*42*, *43*), not as it evolves in time. In this case, the temporal resolution of the UEM remains the same as the temporal profile of the entire train of pulses, which lasts a few hundred femtoseconds to infinite when a continuous wave gating laser is used (*42*, *43*). Therefore, the train of attosecond electron pulses is constrained to imaging periodic dynamics, such as the repeatable oscillation of scattered light (*42*, *43*). However, to resolve nonperiodic attosecond dynamics, such as charge migration, electron-based phase transition, and electronic motion in the solid-state system presented in this work, a single attosecond electron pulse (to confine the probing to a single event) must be generated and used inside the microscope.

In this work, we generate a single attosecond electron pulse by the optical gating approach using a polarization-gated half-cycle laser pulse (*31*, *44*, *45*). Then, we demonstrate the attained attosecond temporal resolution by measuring the attosecond electron diffraction to probe the sub-half-cycle field-driven electron dynamics of multilayer single crystalline graphene (fig. S1). We studied these electron dynamics theoretically and calculated their effect on changing the scattered electron intensity. Then, we compare the simulation results with our attosecond electron diffraction measurements, which show good agreement. The ability to resolve this subcycle dynamics demonstrates the attosecond resolution in the electron microscope and establishes what we coined “attomicroscopy.” The attosecond imaging introduced in this work enables the study of the attosecond electron dynamics of matter and opens the door for a variety of attosecond imaging applications. Moreover, this long-awaited imaging of electron motion in action can reveal electron dynamics in complex and quantum systems and promises to break new ground in physics, chemistry, biochemistry, and biology.

## RESULTS AND DISCUSSION

**Fig. 1.**
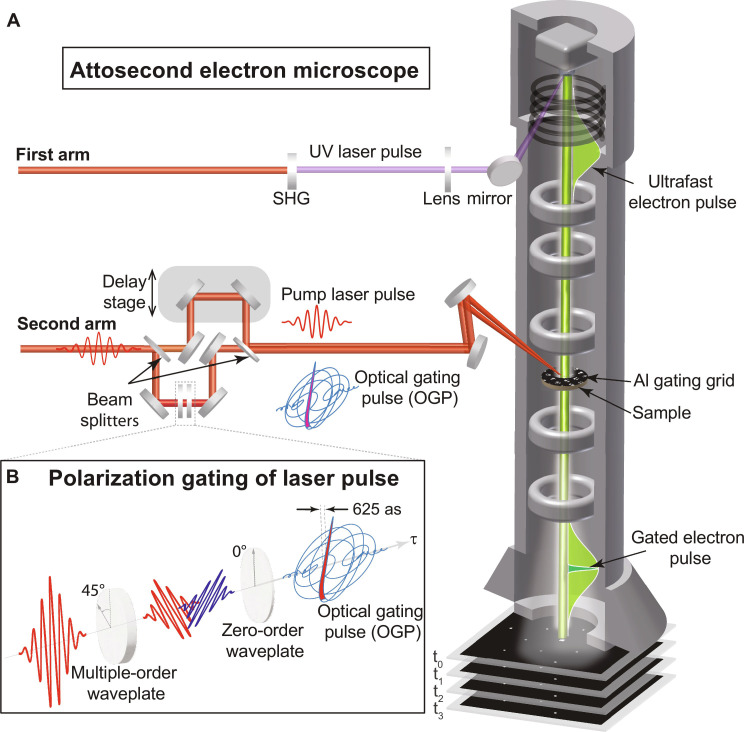
Attosecond electron microscope setup. (**A**) The setup consists of two arms; the first arm is an NIR laser pulse propagating through a second-harmonic generation (SHG) crystal to generate a UV laser pulse. This UV beam is directed to the photocathode inside the microscope to emit ultrafast electron pulses. In the second arm, a 5-fs laser pulse is divided by a beamsplitter into two beams. The first beam is reflected off two mirrors mounted on a nanometer-precision delay stage and is sent to the microscope. This beam is used as the pump pulse to trigger the electron motion in the system under study. The second beam undergoes the PG process, which is illustrated in (**B**). In this process, the input linearly polarized laser pulse passes through multi-order and zero-order waveplates set at 45° and 0°, respectively. The generated pulse has a half-cycle linear-polarized field in the middle and a circularly polarized field at the edges. This pulse is used as an optical gating pulse (OGP) of the electrons inside the microscope. The OGP beam is directed to the sample position where the electron gating and the generation of the attosecond electron pulses take place. These gated electrons can be used to record images (diffraction/direct) to probe the electron dynamics of matter.

**Fig. 2.**
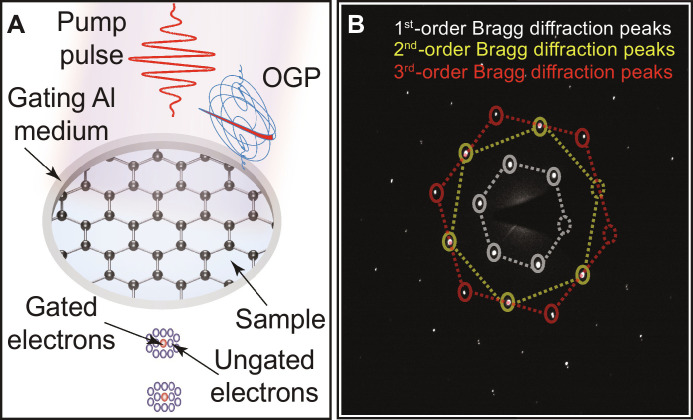
Optical gating of electron pulse. (**A**) Illustration of the electron and laser pulse interaction on the gating medium (aluminum grid) above the sample of graphene. (**B**) Measured diffraction pattern of the multilayer graphene sample.

In our attosecond electron microscopy setup (Fig. 1A), the polarization-gated half-cycle laser pulse has been generated using the polarization gating (PG) method (see the basic principle in Fig. 1B and the setup in fig. S2A). This pulse is used as an optical gating pulse (OGP) to gate and generate the single attosecond electron pulses inside the microscope by the optical gating approach, which can be explained as follows: First, the main ultrafast electron pulse (a few hundred femtoseconds duration) propagates from the photocathode to the transmission electron microscope (TEM) dense-mesh aluminum grid (thickness = 20 ±3 μm and pitch size is 83 μm) placed on the top of the sample (see Fig. 2A), which acts as a gating medium. We opted to use aluminum due to its broad frequency response (*46*). The OGP beam (beam diameter, ~200 μm) is directed to the aluminum grid as illustrated in Fig. 2A. The angle between the electron and the OGP beams is <2° (see fig. S2B). The electron and OGP pulses are traveling at different speeds, so no interaction occurs before the gating medium. Second, the OGP interacts with the gating medium and induces an evanescent scattered light field, which has a comparable speed and interacts with the main electron pulse. The electrons couple efficiently with the linearly polarized part of the scattered field [the interaction with the circularly polarized sides of the field is minimal due to the averaging out of these parts (*41*)] and exchange momentum. Then, some of these electrons gain or lose (*n*) number of photons from the scattered field. Hence, these electrons are gated in a temporal window similar to the duration of the linearly polarized half-cycle of the OGP (625 as) to generate a single-attosecond electron pulse. The generated attosecond electron pulse in our approach is generated on the Al grid just on top of the sample, so the space charge effect does not substantially affect the gated electron pulse duration. The number of gated electrons is estimated to be 0.1% of the total number of electrons (~10^6^ electrons/s) generated from the photocathode, which is sufficient to probe and image the ultrafast electron motion dynamics, enhancing the temporal resolution of the TEM to the subfemtosecond time scale. As a proof-of-principle of the obtained attosecond resolution, the subfemtosecond gated electrons are used to image the sub-half-cycle field-driven electron motion dynamics triggered by the pump pulse (field strength = 2.5 V/nm) in a single-crystal multilayer graphene sample (sample preparation is explained in the Supplementary Materials) by attosecond time-resolved electron diffraction measurements (diffraction pattern of graphene is shown in Fig. 2B).

**Fig. 3.**
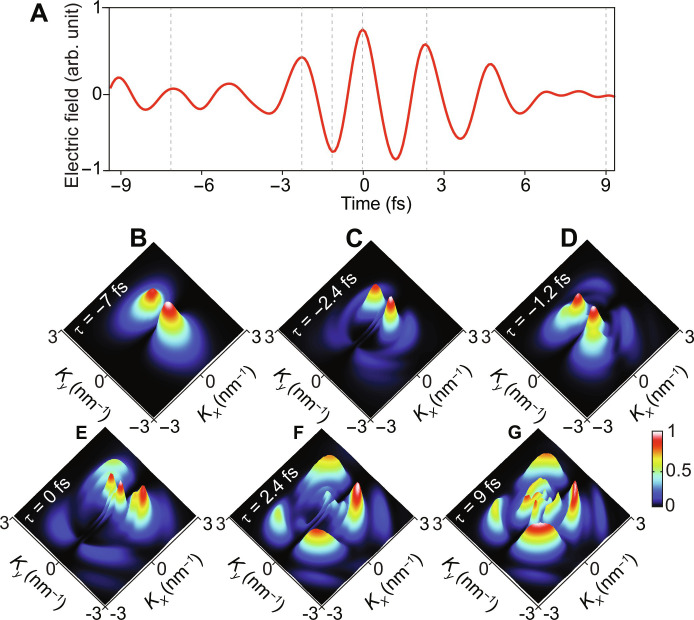
Light field–induced electron dynamics in the reciprocal space of graphene. (**A**) The measured waveform of the pump pulse which is used in the theoretical calculations and diffraction measurements. (**B** to **G**) Snapshots of the EDD in the reciprocal space of graphene at different time instants (indicated by dashed gray lines in (A) in the presence of and after the end of the driver field. Each snapshot is normalized with respect to the snapshot in (G) for better visualizing of the EDD (the normalization factor is mentioned in the right side inset).

Before we discuss our attosecond diffraction experiment results, we performed a theoretical study to understand the electron motion dynamics in graphene. In a strong light field, there are two induced electron dynamics: (i) the interband dynamics caused by the population transfer between the valence band (VB) and conduction band (CB) and the related coherent dynamics, and (ii) the intraband dynamics generated due to the electron motion within the bands (*47*). These field-driven dynamics follow the waveform of the pump pulse and occur in a sub-half-cycle time scale. Accordingly, it was crucial to measure our pump waveform using the all-optical light field sampling methodology we established elsewhere (*7*, *8*, *48*). The sampled field is shown in Fig. 3A. Accordingly, in our presented theoretical study, we used the same parameters of the experiment and the measured pump pulse waveform.

Our theoretical study is based on quantum-mechanical simulations of the field-induced electron motion dynamics in multilayer graphene. It is composed of two main parts:

In the first part, we calculated the evolution of the electron density distribution (EDD) in reciprocal space by solving the Bloch equation (*49*, *50*)

$$\begin{array}{c} i\hbar \frac{\partial }{\partial t}\rho_{m,n}\left(k,t\right)=\left(E_{m}\left(k_{t}\right)-E_{n}\left(k_{t}\right)\right)\rho_{m,n}\left(k,t\right)+\\ E\left(t\right)\cdot {\left\{D\left(k_{t}\right),\rho \left(k,t\right)\right\}}_{m,n}-i\frac{1-\delta_{m,n}}{T_{d}}\rho_{m,n}\left(k,t\right) \end{array}$$ (1)

where ***E***(*t*) is the applied electric field, ρ_m,n_(***k***, *t*) denotes the matrix element of the density matrix ρ(***k***, *t*), *E*_i_(***k***_t_) are the energies of the bands, ***D***(***k***_t_) is the matrix of transition dipole moments, the commutator symbol “{}^”^ is defined as {*A*, *B*} = *AB* − *BA*, and *T*_d_ is the interband dephasing time. The required energies of the bands *E_i_*(***k***) and the transition dipole moments ***D***(***k***) were obtained by employing the nearest-neighbor tight-binding model of *M*-layer (*M* = 6 in our simulations) *AB*-stacked graphene.

The simulations are performed in the time-dependent crystal momentum frame, which evolves according to the Bloch acceleration theorem

kt=k+eℏ At (2)

where ***k*** is the wave vector of the field-free electron and the interaction with the applied electric field ***E***(*t*) (Fig. 3A) is governed by the corresponding vector potential $A\left(t\right)=-\int_{-\infty }^{t}dt\prime E\left(t\prime \right)$ . We assumed the applied electric field is linearly polarized along the C-C bonds of the graphene sample and has a peak strength of 2.5 V/nm.

At the initial moment of time *t*_0_, the electron density occupies the lowest energy electronic state, so that ρ_1,1_(***k***, *t*_0_) = 1 and ρ_*m*,*m*_(***k***, *t*_0_) = 0 for *m* > 1. The action of the laser field leads to the population transfer from the ground state to the excited electronic states of the system, which is reflected in the changes of the matrix elements of the density matrix ρ(***k***, *t*). The evolution of the matrix element ρ_12,12_(***k***, *t*), which represents the part of the electron density occupying the highest electronic state (reciprocal space) of six-layer graphene. The rate of the population transfer for given parameters of the applied field at the specific point in reciprocal space is defined by the transition dipole moments connecting the electronic states but also depends on the energy gaps between the corresponding states (see Eq. 1). The reciprocal lattice of graphene represents a hexagonal pattern with two inequivalent and high-symmetry points *K* and *K*′, referred to as Dirac points (*51*). The VB and CB of graphene touch each other at these points, forming the so-called Dirac cones. While the energy gaps in the vicinity of the crossings become small, the transition dipole moments become large, which make the population transfer very efficient at the Dirac points. The redistribution of populations between the bands reflected in the changes of the diagonal elements ρ_*m*,*m*_(***k***, *t*) of the density matrix, also causes the evolution in time of the off-diagonal elements ρ_*m*,*n*_(***k***, *t*), which are referred to as the electronic coherences.

The calculated snapshots of the EDD dynamics in the reciprocal space at different time (τ) instants (as shown in Fig. 3, B to G), where the EDD around the Dirac point (*K_x_* = 0 and *K_y_* = 0) is driven by the Landau-Zener (LZ) mechanism dynamics in space (*52*). Initially, at the arrival of the field (τ = −7 fs), only the excited electrons localized around the Dirac point are involved in the dynamics, as illustrated in Fig. 3B. Then, as the field strength increases at τ = −2.4 fs, the excited electrons migrate to the positive *K_x_* direction, as depicted in Fig. 3C. At τ = −1.2 fs, the electrons are returned toward the *K_x_* negative direction following the field shape, as shown in Fig. 3D. At the field’s highest half-cycle strength (τ = 0 fs), the electrons reach their largest displacement along *K_x_* axis (see Fig. 3E). The displacement of electrons towards negative *K_x_* (τ = −1.2 fs, Fig. 3D) and positive *K_x_* (τ = 0 fs, Fig. 3C) are not symmetric with respect to the Dirac point, indicating that the electron displacement depends on the strength of the driver field. Moreover, the number of excited electrons at the reciprocal space increases, and the EDD spreads in positive and negative directions of *K_y_*. At τ 2.4 fs, the EDD is minimal around the Dirac point and more spread in both directions (positive and negative values) of *K_x_* and *K_y_*, as shown in Fig. 3F. After the end of the field (at τ = 9 fs); the EDD is all over the reciprocal space (Fig. 3G). While the populations of the bands remain constant after the action of the external field on the system, the electronic coherences are expected to decay rapidly due to the various decoherence effects, such as temperature fluctuations and the variations of the laser field parameters. The full mapping of the electron dynamics in the reciprocal space is provided in movie S1.

The computed reciprocal space density matrix ρ(***k***, *t*) provides access to the real-space EDD [*Q*(***r***, *t*)], which can be decomposed in the noncoherent *Q*^ncoh^(***r***, *t*) and coherent *Q*^coh^(***r***, *t*) contributions as following

$$\begin{array}{c} Q\left(r,t\right)=\frac{2}{S_{\text{BZ}}}\left(\underset{Q^{\text{ncoh}}\left(r,t\right)}{\underbrace{\sum_{m=1}^{2M}\int \rho_{m,m}\left(k,t\right){\left|\Psi_{m}\left(k_{t},t\right)\right|}^{2}dk}}\right.+\\ \left.\underset{Q^{\text{coh}}\left(r,t\right)}{\underbrace{2\sum_{m>n}^{2M}\int \text{Re}\left\{\rho_{m,n}\left(k,t\right)\Psi_{m}^{*}\left(k_{t},r\right)\Psi_{n}(k_{t},r)\right\}dk}}\right) \end{array}$$ (3)

where *S*_BZ_ denotes the area of the Brillouin zone, and $\Psi_{n}\left(k,r\right)=\sum_{j=1}^{2M}\psi_{\mathrm{nj}}\left(k\right)\Phi_{j}\left(k,r\right)$ are the field-free wave functions of π electrons of graphene (in total 12 electronic states for six-layer graphene). The expansion coefficients ψ*_nj_*(***k***) are the eigenstates of the tight-binding Hamiltonian, and Φ*_j_*(***k***, ***r***) are the Bloch states of graphene

$$\Phi_{j}\left(k,r\right)=\frac{1}{\sqrt{N}}\sum_{i=1}^{N}e^{ik\cdot R_{i}}\varphi_{2p_{z}}\left(x-R_{i}\right)$$ (4)

where the summation is made over *N* atoms in the sublattice, and φ_2*p_z_*_ denotes the carbon 2*p_z_* atomic orbitals centered at the nuclei located at the position ***R****_i_*.

**Fig. 4.**
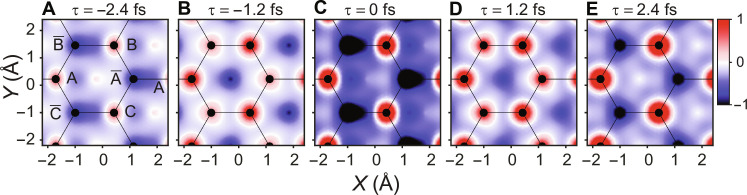
Graphene electron motion dynamics in real space. (**A** to **E**) Calculated snapshots of the field-induced electron motion at different time instants. These snapshots show the change in the EDD around and between the carbon atoms of graphene at different time instants in real space. The red, blue, and white colors represent the high (positive), low (negative), and zero values of the EDD, respectively.

In the second part, we calculated and plotted snapshots of the real space EDD at different time instants in Fig. 4 (a full movie is provided in movie S2). Note that the carbon atoms of the top graphene layer and the bonds between these atoms are shown in solid black circles and lines, respectively. Before the field arrival, the electrons are in the equilibrium state, and no EDD change occurs. At the maxima of the first half-cycle (τ = −2.4 fs), the change in the EDD is observable, as shown in Fig. 4A. In this snapshot, the EDD is more localized around A, B, and C than $\bar{A}$ , $\bar{B}$ , and $\bar{C}$ atoms. Also, the EDD intensities between $\bar{B}B$ , $\bar{C}C$ , and $\bar{\;A}A$ (from the next unit cell) atoms are lower than between $A\bar{B}$ , $A\bar{C}$ , $B\bar{A\;}$ , and $\bar{A}C$ atoms in space.

When the field flips the direction at τ = −1.2 fs (Fig. 4B), the electrons migrate toward the $\bar{A}$ , $\bar{B}$ , and $\bar{C}$ atoms, and the EDD localization differences between A, B, and C and $\bar{A}$ , $\bar{B}$ , and $\bar{C}$ atoms decrease. Furthermore, near the field maxima (τ = 0 fs), the EDD localization difference around and between the atoms is maximized, as illustrated in Fig. 4C. Then, after a half-cycle (at τ = 1.2 fs), the field flips the direction, and the EDD difference around the atoms becomes smaller as shown in Fig. 4D. At τ = 2.4 fs (the maxima of the third half-cycle of the field), the localizing difference of the EDD increase again, as illustrated in Fig. 4E. Although the EDD between the carbon atoms in Fig. 4E remains less than in Fig. 4C and higher than in Fig. 4A because in this case, the field strength is less than the main half cycle at τ = 0 fs but higher than the field strength of the first half cycle at τ = −2.4 fs. This indicates that the EDD in real space depends on the direction and the strength of the driver field, similar to the electron motion in the reciprocal space (Fig. 3, B to G).

Next, we calculated the electron diffraction signal of multilayer graphene based on the simulated EDD [*Q*(***r***, *t*)] dynamics in real space shown in Fig. 4. Our approach is based on the assumption that projectile electrons scatter from the graphene electron density elastically (*53*), e.g., without causing interference effects (*54*). We note in passing that taking the quantum nature of the scattering process into consideration is a notoriously difficult task. Despite the notable efforts in this direction in recent years (*54*–*70*), full quantum calculations are possible only for atoms and small molecules under certain assumptions, while no such calculations for electron dynamics in solid-state systems have been reported so far.

**Fig. 5.**
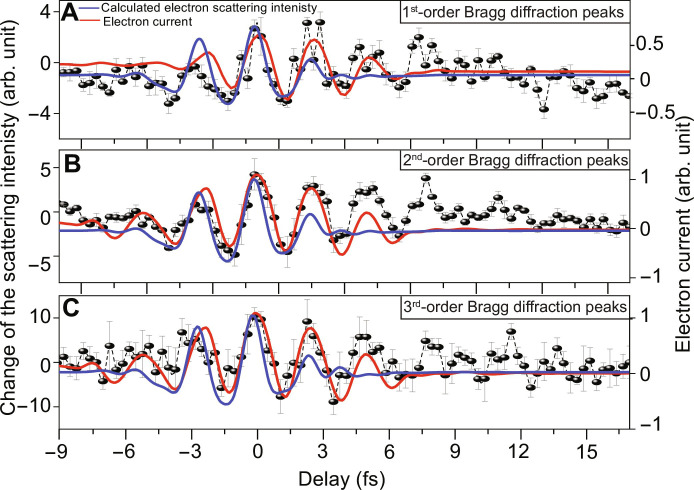
Attosecond electron diffraction of graphene. (**A** to **C**) The measured average scattering intensity changes of the first-, second-, and third-order diffraction peaks as they evolve in time are shown as black dots connected with dashed black lines, respectively. The error bars present the SD uncertainty of the relative scattered peak intensity of seven scans. The calculated diffraction intensity oscillations, caused by the field-induced electron motion in real space, are plotted in a solid blue line, while the fitted field-induced electron current is plotted in a solid red line.

In the first-Born approximation, the intensity of the scattered electron beams from a periodic solid is given by (*71*)

$$I_{G}\left(t\right)\propto \frac{1}{G^{2}}{\left|\int_{\text{unit cell}}Q\left(r,t\right)\text{exp}\left(iG\cdot r\right)dr\right|}^{2}$$ (5)

where the integration is performed in the unit cell and **G** is reciprocal lattice vector. Assuming a perpendicular orientation of the graphene sample with respect to the incoming electron beam and using the fact that $G=\frac{2\pi }{a}\left(p\left(\frac{1}{\sqrt{3}},1\right)+q\left(\frac{1}{\sqrt{3}},-1\right)\right)$ for graphene (*51*), we can integrate Eq. 5 along *z* axis explicitly thus obtaining

$$\begin{array}{c} I_{p,q}\left(t\right)\propto \frac{3a^{2}}{16\left(p^{2}-\mathrm{pq}+q^{2}\right)\pi^{2}}\\ {\left|\int_{\text{unit cell}}Q\left(x,y,t\right)\text{exp}\left(i\frac{2\pi }{a}\left(\frac{1}{\sqrt{3}}\;\left(p+q\right)x+\left(p-q\right)y\right)\right)\text{dxdy}\right|}^{2} \end{array}$$ (6)

where *Q*(*x*, *y*, *t*) = ∫ *Q*(***r***, *t*)*dz* is the electron density integrated over *z* direction and *p* and *q* are Miller indices. The obtained Bragg spots diffraction intensities *I*_*p*,*q*_(*t*) are averaged in each order and convoluted with a Gaussian function to account for a finite duration of the incident electron beam

$${\tilde{I}}_{\text{BZ}}\;\left(t\right)=\int_{-\infty }^{+\infty }\frac{1}{\sigma \sqrt{2\pi }}\text{exp}\left[-\frac{{\left(\tau -t\right)}^{2}}{2\sigma^{2}}\right]\sum_{p,q\in \text{BZ}}I_{p,q}\left(\tau \right)\;d\tau$$ (7)

where the summation is performed over all combinations of indices belonging to a corresponding order, and parameter $\sigma =\frac{\text{FWHM}}{\sigma \sqrt{2\pi }}$ characterizes the width of the electron pulse. The calculated scattered intensity changes for the first, second and third Bragg diffraction peaks are shown in blue color in Fig. 5 (A to C, respectively).

The change in the scattered electron intensity is attributed to the displacement of the EDD between the carbon atoms in real space (Fig. 4), which is caused mainly by intraband dynamics. While the EDD change due to the interband dynamics is localized around each carbon atom and occurs in the same direction as the incident electron beam. Hence, the interband transition can barely affect the scattering electron intensity.

In our experiment, the electron diffraction pattern of graphene (Fig. 2B) is recorded as a function of the time delay (with a time step size of 300 as) between the OGP (gated electron pulses) and the pump pulses. Note the OGP is set at a low value of ~0.85 V/nm to ensure that it is not triggering the graphene electron dynamics.

The retrieved time-resolved attosecond electron diffraction results show that the intensity of the first-, second-, and third-order scattered diffraction peaks, presented in black dots connected with dashed black lines (average of seven scans) in Fig. 5 (A to C), are modulating as a function of time, respectively. Please note that the gated electrons have subfemtosecond resolution, while the ungated electron temporal resolution remains a few hundred femtoseconds. So, the intensity modulation signature is carried only on the gated electrons, while the ungated electron remains unchanged. Accordingly, this scattered gated electron intensity modulation is retrieved by subtracting the dark diffraction pattern (at τ ≤ −9 fs) and the ungated electrons (background), as explained in the Supplementary Materials. The measured and simulated diffraction change of graphene dynamics triggered by strong field (Fig. 5, A to C) are in acceptable agreement.

Next, to confirm that the main cause for the scattering electron intensity change is the electron motion within the CB as explained earlier, we calculated the intraband

$$J^{\text{intra}}\left(t\right)\propto \sum_{m=1}^{2M}\int \rho_{m,m}\left(k,t\right)\nabla_{k}E_{m}\left(k_{t}\right)dk$$ (8)

and interband band

$$J^{\text{inter}}\left(t\right)\propto 2\sum_{m>n}^{2M}\int Im\left\{\rho_{m,n}\left(k,t\right)\left(E_{m}\left(k_{t}\right)-E_{n}\left(k_{t}\right)\right)D_{n,m}\left(k_{t}\right)\right\}dk$$ (9)

currents and fitted them to the measured diffraction intensity modulations. The fitted curves [plotted in the red line in Fig. 5 (A to C)] follow the main diffraction intensity oscillations of the first-, second-, and third-order peaks. The small deviation on the sides may be attributed to the underestimation of the dispersion effect experienced by the pump field with respect to the sample position in the performed field sampling measurement.

The fitting results show that the contribution of the intraband current is more substantial (estimated to be ~87, ~85, and ~80% for the first, second, and third diffraction peaks’ dynamics, respectively) than the interband current.

The resolution of the half-cycle electron diffraction modulations in the diffraction measurements presented in Fig. 5 (A to C) validates the attained attosecond imaging resolution.

Although our microscope is not equipped with an energy analyzer and spectrometer to directly separate and measure the gated pulse duration, but we performed a few additional experiments to confirm the achieved attosecond resolution, which is the main goal of this work. First, after recording the measurements presented in Fig. 5, we reduced the pump field strength to <2 V/nm (low-field regime) (*47*). The electron diffraction intensity modulation disappeared, indicating that the measured signal is due to graphene’s strong field-driven electron dynamics (see fig. S3). In addition, this result excludes the possibility that the measured diffraction change is increased due to any interference effect between the pump and the gating laser pulses. We observed the scattering intensity modulation signal again by ramping up the power of the pump pulse back to the high field strength of 2.6 V/nm.

In a second confirmation experiment, we blocked the OGP and repeated the electron diffraction measurements. The measured diffraction patterns showed no intensity change in the Bragg spots (at any order) in the time window between −10 and 15 fs, as expected (see fig. S4). In this case, the electron imaging resolution is defined by the main electron pulse from the photocathode (no gated electrons), which is on the order of a few hundred femtoseconds. Then, we released the OGP to gate the electrons again on the sample and repeated the time-resolved diffraction measurement under the same conditions. The intensity oscillations of the diffraction peaks were observed again. Last, we performed the experiment under the same conditions but with the gating medium (aluminum grid) removed. The results show no resolved oscillations (see fig. S5), confirming the measured diffraction results in Fig. 5 (A to C) occurred due to the generation of the subfemtosecond gated electron pulse at the aluminum grid.

Notable, many experimental factors are vital for performing the presented attosecond electron imaging experiment, such as (i) the high repetition rate of the laser, which allows the gating of a high number of electrons, (ii) the high stability of the laser beam intensity (root mean square = 0.3%), which allows us to achieve a low noise level of the electron intensity fluctuation in the microscope, and (iii) the low jitter between the gated electrons (controlled by the OGP pulse) and laser pump pulses, which is measured and determined during our measurements to be in the range of ~100 as. This inherited extremely low jitter is one of the notable features of the demonstrated optical gating approach, which overcomes one of the long-standing challenges in ultrafast time-resolved electron imaging measurements in UEM and ultrafast electron diffraction (UED).

This work demonstrates the attosecond temporal resolution, obtained by attosecond optical gating, inside TEM. Moreover, we performed attosecond electron diffraction imaging to study the electron dynamics of matter. Our results show a strong correlation between field-induced electron dynamics in the CB and the real space electron motion between the carbon atoms in graphene. This work paves the way for recording images of electron motion in the four dimensions of space and time and opens a window to see the quantum world in real systems and answer fundamental questions in physics. Moreover, the capability of attosecond electron microscope imaging to connect the light-induced electron dynamics and the morphology of matter in real-space builds the desired bridge between science and technology to engineer petahertz and quantum photonics (*72*, *73*). Furthermore, electron imaging would allow filming and controlling the chemical and biochemical reactions in real time, which would advance the fields of material synthesis, drug design, and personalized medicine to the next level.

## MATERIALS AND METHODS

In our setup (Fig. 1A), a 1-mJ laser pulse (16 fs) centered at 750 nm is generated from an optical parametric chirped-pulse amplification–based (passively carrier-envelope phase stabilized) laser system with a 20-kHz repetition rate. The beam is divided into two beams by a 10:90 beamsplitter. Each beam is sent to one arm of the setup. In the first arm, the 10% (~2 W) portion of the main laser beam is sent to a second-harmonic generation (SHG) unit to generate an ultraviolet beam (λ = 380 nm). The ultraviolet beam is focused onto a photocathode inside the microscope to generate the main ultrafast electron pulses (typically having a duration of a few hundred femtoseconds). The UV beam is kept at the lowest power to minimize the number of electrons per pulse and the related space-charge effect. Then, these electrons are accelerated to 200 keV and focused by the magnetic lenses into the sample understudy inside the microscope.

In the second arm, 90% of the main laser beam is focused on a hollow-core fiber filled with neon gas under 2.5-bar pressure using a 1-m lens. The beam undergoes a strong nonlinear propagation and generates a broadband spectrum that spans over 250 to 1000 nm. The power of the supercontinuum after the fiber is 4 W (200 μJ). This supercontinuum enters a chirp mirror compressor (optimized to compress a broadband spectrum from 400 to 1000 nm) to generate a Fourier-limited 5-fs laser pulse (p-polarized). Note that the compressor precompensates for the pulse dispersion due to the propagation through the dispersive elements [waveplates, windows, neutral density (ND) filters, etc.] in the beam path until the pulse reaches the sample inside the microscope. The beam exits the compressor and enters the PG unit to generate a polarization-gated laser pulse (fig. S2A). The 5-fs (p-polarized) laser pulse is divided into two beams via a 30:70 beam splitter (BS#1). The transmitted beam carries 70% of the beam power and acts as the “pump” pulse to trigger the ultrafast electron dynamics of the sample. This pump beam also passes through an ND to control the pump pulse field strength. The reflected beam (carries 30% of the beam power) is acting as the OGP. This beam passes through another ND filter to control the OGP power. Then, the beam passes through a multi-order waveplate. Hence, the output beam contains p-polarized and s-polarized components. The multi-order waveplate (thickness = 180 μm) introduces a temporal delay (~4 fs) between the two light polarized components along the ordinary and extraordinary axes of the plate. The angle between the axes of the plate and the incident polarization direction is set to 45°. Hence, the pulse at the output of the waveplate is linearly polarized on the leading and trailing edges (with crossed polarization directions) and it is circularly polarized in the center. Next, this beam propagated through a zero-order quarter waveplate, with the axis forming a 0° angle with respect to the polarization direction of the incident beam. This second waveplate transforms the linear polarization into circular and vice versa; thus, the output pulse has a linearly polarized half-cycle in the middle, and the edges of the pulse are circularly polarized. The linear half-cycle in OGP has an estimated full width at half-maximum (FWHM) of 625 as. Hence, a short temporal gate (<1 fs) of the linearly polarized half-cycle field is obtained to temporally gate the free electron pulses inside the microscope by the optical gating approach (*13*, *31*, *45*). At the exit of the PG setup, both OGP and the pump beams are combined by a second beamsplitter (BS#2) and collinearly propagate to the sample understudy inside the microscope. The delay between the OGP and the electron pulse is controlled to temporally overlap both pulses by a linear stage implemented in the UV laser beam path. On another note, the delay between the pump laser pulse and the OGP is controlled by a high-precise piezo stage with a few nanometers (10 as) resolution introduced in the pump beam path.

## Supplementary Materials

### This PDF file includes:

Supplementary Text Figs. S1 to S5 Legends for movies S1 and S2 References
