# Supplementary material for: Attosecond electron microscopy and diffraction
Source: Sci Adv. 2024 Aug 21;10(34):eadp5805. doi: 10.1126/sciadv.adp5805 (PMC11338230; doi:10.1126/sciadv.adp5805)
Supplement: Supplementary file 1 — Supplementary Text Figs. S1 to S5 Legends for movies S1 and S2 References [file sciadv.adp5805_sm.pdf]

Science Advances
AAAS

Supplementary Materials for

Attosecond electron microscopy and diffraction

Dandan Hui *et al.*

Corresponding author: Mohammed Th. Hassan, mohammedhassan@arizona.edu

Sci. Adv. **10**, eadp5805 (2024)
DOI: 10.1126/sciadv.adp5805

The PDF file includes:

Supplementary Text
Figs. S1 to S5
Legends for movies S1 and S2
References

Other Supplementary Material for this manuscript includes the following:

Movies S1 and S2

Multilayer graphene sample preparation

The single-crystalline few-layer (~6 layers) graphene samples were prepared by mechanical exfoliation from ~2-3 mm high quality natural graphite crystal. A piece of single-crystalline graphite was repeatedly exfoliated with scotch tape down to the thickness of ~2-3 nm. Then, the sample was transferred to a 1000 mesh transmission electron microscope (TEM) grid for measurements. The number of the layers is defined by the light transmittance analysis of the free-standing samples using bright white light. The measured absorption of our sample (shown in Fig. S**1**) is in the range of 14%. The absorption per layer of graphene is reported to be 2.3% (*74*). So, the number of layers in our sample is estimated to be 6 layers.

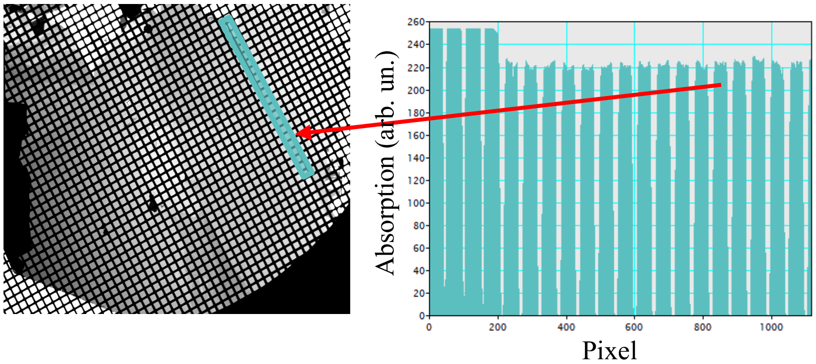

Fig. S1. The characterization of multilayer graphene sample. The TEM image of the sample is shown on the left while the light absorption measurements is shown in on the right. The number of the layers is determined to be ~6 layers.

Time-resolved electron diffraction data analysis

To retrieve the change of the diffraction intensity in different orders as a function of time (shown in Fig. 5(A-C)), the recorded diffraction images at different instants of pump-probe delay time are analyzed as follows:

1- First, we apply median filtering to reduce noise of the acquired diffraction image.
2- Each diffraction peak intensity is the summation over a selected Region-of-interest (ROI). The ROI position is centered at the peak's maximum intensity, and the ROI size is chosen as twice of the peak size.
3- The summed intensity of each peak is then normalized to a summation of the intensities over a certain area of the main beam leakage, to compensate for the main electron beam intensity fluctuation. Therefore, we can get the figure of normalized peak intensities versus time.
4- For each peak, the intensity is again normalized to the average intensity of the points before the field arrival ($t \leq -9fs$) and subtract the dark diffraction pattern (which includes the ungated electrons). Hence, the intensity change of the gated electrons is retrieved and plotted in Fig. 5.

Fitting process

We fitted the calculated intraband current ($\boldsymbol{J}^{\mathrm{intra}}$) and interband current ($\boldsymbol{J}^{\mathrm{inter}}$) to the measured 1st-, 2nd-, and 3rd-order electron scattering oscillations individually using the following function:

$$y\,(t) = d + c\big[a * \boldsymbol{J}^{\mathrm{intra}}(t) + b * \boldsymbol{J}^{\mathrm{inter}}(t)\big]$$

where a and b are the amplitudes of the intraband and interband currents, respectively, c is the overall scale of the total current, and d is the shift value.

The fitting procedure is based on the derivative-free Nelder-Mead simplex algorithm18 which is used to find the minimum of a multivariable function $f(t)$

$$\min_{a,b,c,d}\{f(t)\} = I^{\exp}(t) - y(t),$$

where $I^{\exp}(t)$ is the experimentally measured scattering intensities, and the optimization is performed with respect to the a, b, c, d parameters. The algorithm constructs a simplex of n + 1 points for n-dimensional vector of initial parameters (4 parameters in our case). Each point in the simplex is computed by adding 5% to each component of the parameters vector. The algorithm uses these n points as elements of the simplex in addition to the central point. Accordingly, the best value of the constructed simplex is used as the central point for the next iteration of the algorithm until convergence. The fitting outputs a vector of parameters among which the values of a and b indicate the contribution ratios of the intraband and interband currents, respectively, to the measured diffraction intensity.

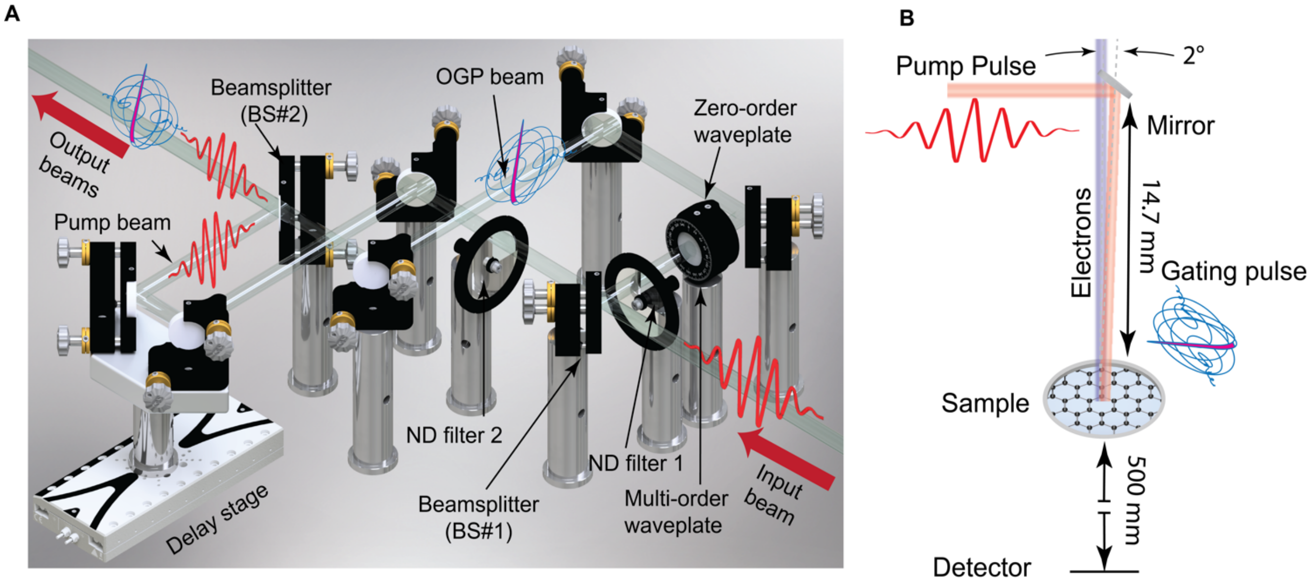

Fig. S2. The Polarization Gating (PG) setup. (A) the optical setup to generate the polarization gating laser pulse. (B) The p-polarized pump and polarization gated (laser gating) pulses are colinearly propagate and directed by a mirror inside the microscope to the sample. The electron beam coming from the photocathode and illuminates the sample. The angle between the laser and electron beams is small angle (about 2°). Then, the scattered electron beam is collected on the detector.

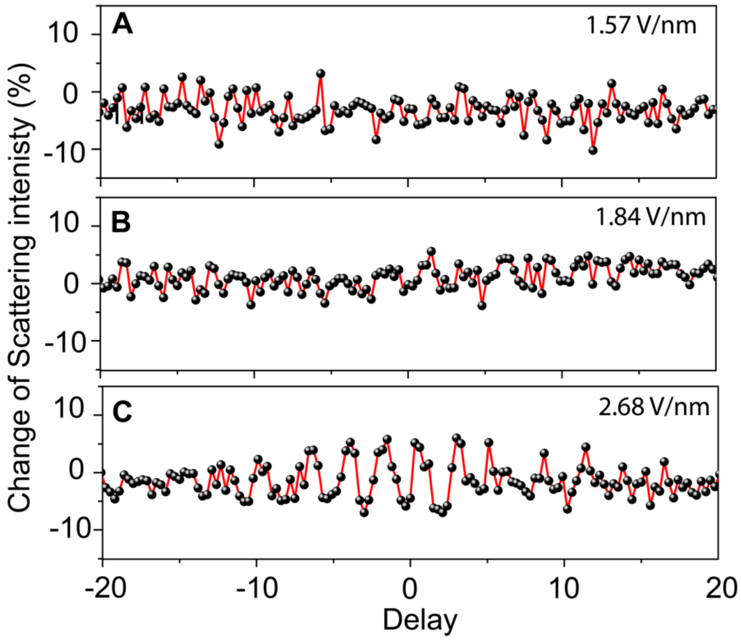

Fig. S3: The measured time-resolved diffraction of graphene at different pump field strength (A) 1.57, **(B)** 1.84**, (C)** 2.68 V/nm.

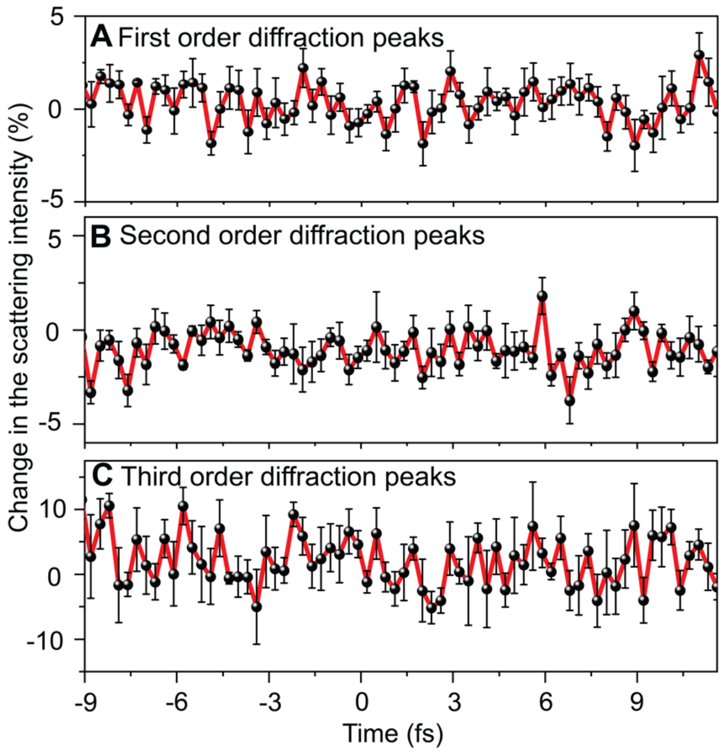

Fig. S4: The time resolved diffraction measurement when the sample is illuminated only with the pump beam and the OGP is blocked. (**A**), (**B**), and (**C**) are the scattering intensity changes for the first, second and third-order diffraction peaks, respectively. The results show no intensity oscillations which indicate that the pump laser is not changing the number of electrons.

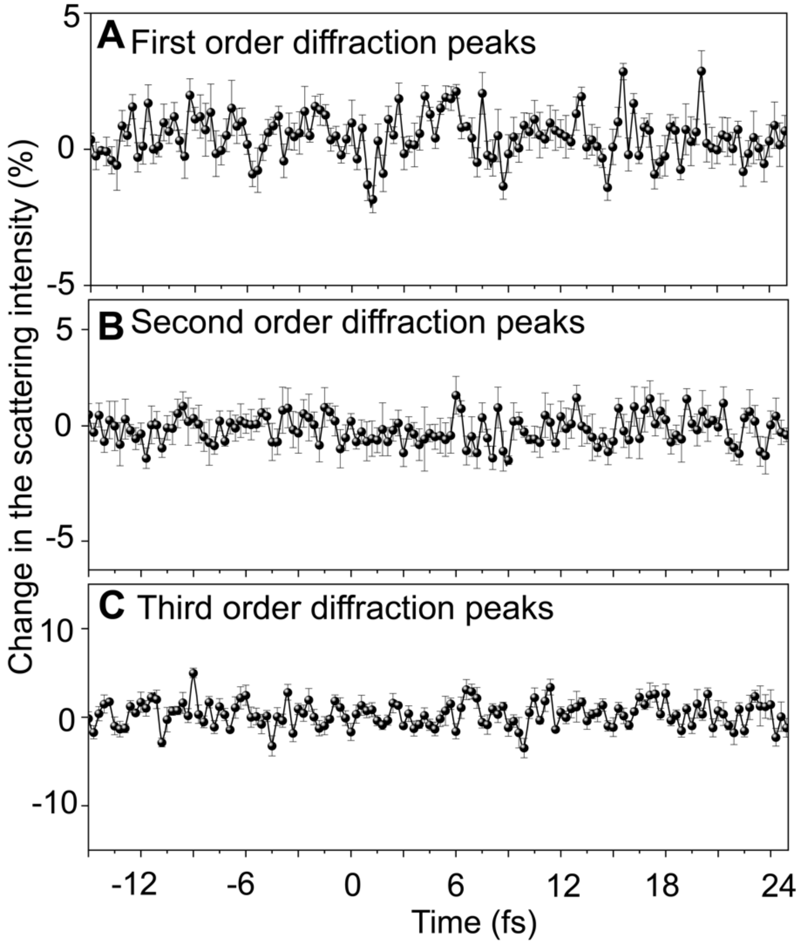

Fig. S5: The time resolved diffraction measurement when the sample is illuminated with the pump beam and the OGP but with no gating medium. (A), (B), and (C) show no scattering intensity changes for the first, second and third-order diffraction peaks, respectively.

Movie Captions

Movie 1: The filed-induced electron motion dynamics in graphene reciprocal space.

Movie 2: The electron motion dynamics between the carbon atoms of graphene in real space.

REFERENCES AND NOTES

1. F. Krausz, *Electrons in Motion: Attosecond Physics Explores Fastest Dynamics*. (World Scientific Publishing Company Pte, Limited, 2019).

2. P. B. Corkum, F. Krausz, Attosecond science. *Nat. Phys.* **3**, 381–387 (2007).

3. F. Krausz, M. Ivanov, Attosecond physics. *Rev. Mod. Phys.* **81**, 163–234 (2009).

4. F. Calegari, D. Ayuso, A. Trabattoni, L. Belshaw, S. De Camillis, S. Anumula, F. Frassetto, L. Poletto, A. Palacios, P. Decleva, J. B. Greenwood, F. Martín, M. Nisoli, Ultrafast electron dynamics in phenylalanine initiated by attosecond pulses. *Science* **346**, 336–339 (2014).

5. G. Sansone, F. Kelkensberg, J. Pérez-Torres, F. Morales, M. F. Kling, W. Siu, O. Ghafur, P. Johnsson, M. Swoboda, E. Benedetti, F. Ferrari, F. Lépine, J. L. Sanz-Vicario, S. Zherebtsov, I. Znakovskaya, A. L'Huillier, M. Y. Ivanov, M. Nisoli, F. Martín, M. J. J. Vrakking, Electron localization following attosecond molecular photoionization. *Nature* **465**, 763–766 (2010).

6. F. Calegari, G. Sansone, S. Stagira, C. Vozzi, M. Nisoli, Advances in attosecond science. *J. Phys. B At. Mol. Opt. Phys.* **49**, 062001 (2016).

7. D. Hui, H. Alqattan, S. Yamada, V. Pervak, K. Yabana, M. T. Hassan, Attosecond electron motion control in dielectric. *Nat. Photonics* **16**, 33–37 (2022).

8. H. Alqattan, D. Hui, M. Sennary, M. T. Hassan, Attosecond electronic delay response in dielectric materials. *Faraday Discuss.* **237**, 317–326 (2022).

9. B. Wolter, M. G. Pullen, A.-T. Le, M. Baudisch, K. Doblhoff-Dier, A. Senftleben, M. Hemmer, C. D. Schröter, J. Ullrich, T. Pfeifer, R. Moshammer, S. Gräfe, O. Vendrell, C. D. Lin, J. Biegert, Ultrafast electron diffraction imaging of bond breaking in di-ionized acetylene. *Science* **354**, 308–312 (2016).

10. J. Duris, S. Li, T. Driver, E. G. Champenois, J. P. MacArthur, A. A. Lutman, Z. Zhang, P. Rosenberger, J. W. Aldrich, R. Coffee, G. Coslovich, F.-J. Decker, J. M. Glownia, G. Hartmann, W. Helml, A. Kamalov, J. Knurr, J. Krzywinski, M.-F. Lin, J. P. Marangos, M. Nantel, A. Natan, J. T. O'Neal, N. Shivaram, P. Walter, A. L. Wang, J. J. Welch, T. J. A. Wolf, J. Z. Xu, M. F. Kling, P. H. Bucksbaum, A.

Zholents, Z. Huang, J. P. Cryan, A. Marinelli, Tunable isolated attosecond X-ray pulses with gigawatt peak power from a free-electron laser. *Nat. Photonics* **14**, 30–36 (2020).

11. M. Garg, K. Kern, Attosecond coherent manipulation of electrons in tunneling microscopy. *Science* **367**, 411–415. (2020).

12. A. H. Zewail, *4D Visualization of Matter*. (Imperial College Press, 2014).

13. M. T. Hassan, Attomicroscopy: From femtosecond to attosecond electron microscopy. *J. Phys. B: At. Mol. Opt. Phys.* **51**, 032005 (2018).

14. A. Arbouet, G. M. Caruso, F. Houdellier, Ultrafast transmission electron microscopy: Historical development, instrumentation, and applications. *Adv. Imaging Electron Phys.* **207**, 1–72 (2018).

15. W. E. King, G. H. Campbell, A. Frank, B. Reed, J. F. Schmerge, B. J. Siwick, B. C. Stuart, P. M. Weber, Ultrafast electron microscopy in materials science, biology, and chemistry. *J. Appl. Phys.* **97**, 111101–111127 (2005).

16. A. Adhikari, J. K. Eliason, J. Sun, R. Bose, D. J. Flannigan, O. F. Mohammed, Four-dimensional ultrafast electron microscopy: Insights into an emerging technique. *ACS Appl. Mater. Interfaces* **9**, 3–16 (2017).

17. O. Bostanjoglo, R. Elschner, Z. Mao, T. Nink, M. Weingärtner, Nanosecond electron microscopes. *Ultramicroscopy* **81**, 141–147 (2000).

18. B. Barwick, H. S. Park, O.-H. Kwon, J. S. Baskin, A. H. Zewail, 4D imaging of transient structures and morphologies in ultrafast electron microscopy. *Science* **322**, 1227–1231 (2008).

19. H. W. Kim, N. A. Vinokurov, I. H. Baek, K. Y. Oang, M. H. Kim, Y. C. Kim, K.-H. Jang, K. Lee, S. H. Park, S. Park, J. Shin, J. Kim, F. Rotermund, S. Cho, T. Feurer, Y. U. Jeong, Towards jitter-free ultrafast electron diffraction technology. *Nat. Photonics* **14**, 245–249 (2020).

20. J. Yang, X. Zhu, T. J. A. Wolf, Z. Li, J. P. F. Nunes, R. Coffee, J. P. Cryan, M. Gühr, K. Hegazy, T. F. Heinz, K. Jobe, R. Li, X. Shen, T. Veccione, S. Weathersby, K. J. Wilkin, C. Yoneda, Q. Zheng, T. J.

Martinez, M. Centurion, X. Wang, Imaging CF3I conical intersection and photodissociation dynamics with ultrafast electron diffraction. *Science* **361**, 64–67 (2018).

21. B. J. Siwick, J. R. Dwyer, R. E. Jordan, R. D. Miller, Ultrafast electron optics: Propagation dynamics of femtosecond electron packets. *J. Appl. Phys.* **92**, 1643–1648 (2002).

22. G. Sciaini, R. D. Miller, Femtosecond electron diffraction: Heralding the era of atomically resolved dynamics. *Rep. Prog. Phys.* **74**, 096101 (2011).

23. H. Ihee, V. A. Lobastov, U. M. Gomez, B. M. Goodson, R. Srinivasan, C.-Y. Ruan, A. H. Zewail, Direct imaging of transient molecular structures with ultrafast diffraction. *Science* **291**, 458–462 (2001).

24. B. J. Siwick, J. R. Dwyer, R. E. Jordan, R. D. Miller, An atomic-level view of melting using femtosecond electron diffraction. *Science* **302**, 1382–1385 (2003).

25. R. Ernstorfer, M. Harb, C. T. Hebeisen, G. Sciaini, T. Dartigalongue, R. D. Miller, The formation of warm dense matter: Experimental evidence for electronic bond hardening in gold. *Science* **323**, 1033–1037 (2009).

26. R. P. Chatelain, V. R. Morrison, C. Godbout, B. J. Siwick, Ultrafast electron diffraction with radio-frequency compressed electron pulses. *Appl. Phys. Lett.* **101**, 081901 (2012).

27. M. Gao, C. Lu, H. Jean-Ruel, L. C. Liu, A. Marx, K. Onda, S.-Y. Koshihara, Y. Nakano, X. Shao, T. Hiramatsu, G. Saito, H. Yamochi, R. R. Cooney, G. Moriena, G. Sciaini, R. J. Dwayne Miller, Mapping molecular motions leading to charge delocalization with ultrabright electrons. *Nature* **496**, 343–346 (2013).

28. T van Oudheusden, P. L. E. M Pasmans, S. B. van der Geer, M. J. de Loos, M. J. van der Wiel, O. J. Luiten, Compression of subrelativistic space-charge-dominated electron bunches for single-shot femtosecond electron diffraction. *Phys. Rev. Lett.* **105**, 264801 (2010).

29. V. R. Morrison, R. P. Chatelain, K. L. Tiwari, A. Hendaoui, A. Bruhács, M. Chaker, B. J. Siwick, A photoinduced metal-like phase of monoclinic VO2 revealed by ultrafast electron diffraction. *Science* **346**, 445–448 (2014).

30. A. Feist, K. E. Echternkamp, J. Schauss, S. V. Yalunin, S. Schäfer, C. Ropers, Quantum coherent optical phase modulation in an ultrafast transmission electron microscope. *Nature* **521**, 200–203 (2015).

31. M. Th. Hassan, J. S. Baskin, B. Liao, A. H. Zewail, High-temporal-resolution electron microscopy for imaging ultrafast electron dynamics. *Nat. Photonics* **11**, 425–430 (2017).

32. K. E. Priebe, C. Rathje, S. V. Yalunin, T. Hohage, A. Feist, S. Schäfer, C. Ropers, Attosecond electron pulse trains and quantum state reconstruction in ultrafast transmission electron microscopy. *Nat. Photonics* **11**, 793–797 (2017).

33. M. R. Otto, L. P. René de Cotret, M. J. Stern, B. J. Siwick, Solving the jitter problem in microwave compressed ultrafast electron diffraction instruments: Robust sub-50 fs cavity-laser phase stabilization. *Struct. Dyn.* **4**, 051101 (2017).

34. A. Ryabov, J. W. Thurner, D. Nabben, M. V. Tsarev, P. Baum, Attosecond metrology in a continuous-beam transmission electron microscope. *Sci. Adv.* **6**, eabb1393 (2020).

35. M. Kozák, J. McNeur, K. J. Leedle, H. Deng, N. Schönenberger, A. Ruehl, I. Hartl, J. Harris, R. L. Byer, P. Hommelhoff, Optical gating and streaking of free electrons with sub-optical cycle precision. *Nat. Commun.* **8**, 14342 (2017).

36. Y. Morimoto, P. Baum, Diffraction and microscopy with attosecond electron pulse trains. *Nat. Phys.* **14**, 252–256 (2018).

37. C. M. S. Sears, E. Colby, R. Ischebeck, C. McGuinness, J. Nelson, R. Noble, R. H. Siemann, J. Spencer, D. Walz, T. Plettner, R. L. Byer, Production and characterization of attosecond electron bunch trains. *Phys. Rev. Spec. Top.–Accel. Beams* **11**, 061301 (2008).

38. G. M. Caruso, F. Houdellier, P. Abeilhou, A. Arbouet, Development of an ultrafast electron source based on a cold-field emission gun for ultrafast coherent TEM. *Appl. Phys. Lett.* **111**, 023101 (2017).

39. S. Borrelli, S. T. Kempers, P. H. A. Mutsaers, O. J. Luiten, in *Structural Dynamics with X-ray and Electron Scattering,* K. Amini, A. Rouzée, M. J. J. Vrakking, Eds. (Royal Society of Chemistry, 2023), vol. 25, pp. 0.

40. B. Barwick, D. J. Flannigan, A. H. Zewail, Photon-induced near-field electron microscopy. *Nature* **462**, 902–906 (2009).

41. G. M. Vanacore, G. Berruto, I. Madan, E. Pomarico, P. Biagioni, R. Lamb, D. McGrouther, O. Reinhardt, I. Kaminer, B. Barwick, H. Larocque, V. Grillo, E. Karimi, F. J. García de Abajo, F. Carbone, Ultrafast generation and control of an electron vortex beam via chiral plasmonic near fields. *Nat. Mater.* **18**, 573–579 (2019).

42. J. H. Gaida, H. Lourenço-Martins, M. Sivis, T. Rittmann, A. Feist, F. J. García de Abajo, C. Ropers, Attosecond electron microscopy by free-electron homodyne detection. *Nat. Photonics*, **18**, 509–515 (2024).

43. D. Nabben, J. Kuttruff, L. Stolz, A. Ryabov, P. Baum, Attosecond electron microscopy of sub-cycle optical dynamics. *Nature,* **619** 63–67 (2023).

44. G. Sansone, E. Benedetti, F. Calegari, C. Vozzi, L. Avaldi, R. Flammini, L. Poletto, P. Villoresi, C. Altucci, R. Velotta, S. Stagira, S. De Silvestri, M. Nisoli, Isolated single-cycle attosecond pulses. *Science* **314**, 443–446 (2006).

45. M. T. Hassan, H. Liu, J. S. Baskin, A. H. Zewail, Photon gating in four-dimensional ultrafast electron microscopy. *Proc. Natl. Acad. Sci. U.S.A.* **112**, 12944–12949 (2015).

46. M. I. Stockman, Nanoplasmonics: Past, present, and glimpse into future. *Opt. Express* **19**, 22029–22106 (2011).

47. T. Higuchi, C. Heide, K. Ullmann, H. B. Weber, P. Hommelhoff, Light-field-driven currents in graphene. *Nature* **550**, 224–228 (2017).

48. H. Alqattan, D. Hui, V. Pervak, M. T. Hassan, Attosecond light field synthesis. *APL Photonics* **7**, 041301 (2022).

49. J. B. Krieger, G. J. Iafrate, Time evolution of Bloch electrons in a homogeneous electric field. *Phys. Rev. B.* **33**, 5494–5500 (1986).

50. C. Liu, Y. Zheng, Z. Zeng, R. Li, Driving-laser ellipticity dependence of high-order harmonic generation in graphene. *Phys. Rev. A* **97**, 063412 (2018).

51. A. H. Castro Neto, F. Guinea, N. M. R. Peres, K. S. Novoselov, A. K. Geim, The electronic properties of graphene. *Rev. Mod. Phys.* **81**, 109–162 (2009).

52. S. N. Shevchenko, S. Ashhab, F. Nori, Landau–zener–stückelberg interferometry. *Phys. Rep.* **492**, 1–30 (2010).

53. V. S. Yakovlev, M. I. Stockman, F. Krausz, P. Baum, Atomic-scale diffractive imaging of sub-cycle electron dynamics in condensed matter. *Sci. Rep.* **5**, 14581 (2015).

54. G. Dixit, O. Vendrell, R. Santra, Imaging electronic quantum motion with light. *Proc. Natl. Acad. Sci. U.S.A.* **109**, 11636–11640 (2012).

55. G. Dixit, R. Santra, Role of electron-electron interference in ultrafast time-resolved imaging of electronic wavepackets. *J. Chem. Phys.* **138**, 134311 (2013).

56. G. Dixit, J. M. Slowik, R. Santra, Proposed imaging of the ultrafast electronic motion in samples using X-ray phase contrast. *Phys. Rev. Lett.* **110**, 137403 (2013).

57. H.-C. Shao, A. F. Starace, Imaging coherent electronic motion in atoms by ultrafast electron diffraction. *Phys. Rev. A* **88**, 062711 (2013).

58. H.-C. Shao, A. F. Starace, Imaging electronic motions in atoms by energy-resolved ultrafast electron diffraction. *Phys. Rev. A* **90**, 032710 (2014).

59. H.-C. Shao, A. F. Starace, Detecting electron motion in atoms and molecules. *Phys. Rev. Lett.* **105**, 263201 (2010).

60. G. Dixit, J. M. Slowik, R. Santra, Theory of time-resolved nonresonant x-ray scattering for imaging ultrafast coherent electron motion. *Phys. Rev. A* **89**, 043409 (2014).

61. G. Dixit, R. Santra, Time-resolved ultrafast x-ray scattering from an incoherent electronic mixture. *Phys. Rev. A* **96**, 053413 (2017).

62. M. Grosser, J. M. Slowik, R. Santra, Attosecond x-ray scattering from a particle-hole wave packet. *Phys. Rev. A* **95**, 062107 (2017).

63. M. Kowalewski, K. Bennett, S. Mukamel, Monitoring nonadiabatic avoided crossing dynamics in molecules by ultrafast X-ray diffraction. *Struct. Dyn.* **4**, 054101 (2017).

64. K. Bennett, M. Kowalewski, J. R. Rouxel, S. Mukamel, Monitoring molecular nonadiabatic dynamics with femtosecond X-ray diffraction. *Proc. Natl. Acad. Sci. U.S.A.* **115**, 6538–6547 (2018).

65. M. Simmermacher, A. Moreno Carrascosa, N. E Henriksen, K. B Møller, A. Kirrander, Theory of ultrafast x-ray scattering by molecules in the gas phase. *J. Chem. Phys.* **151**, 174302 (2019).

66. G. Hermann, V. Pohl, G. Dixit, J. C. Tremblay, Probing electronic fluxes via time-resolved X-ray scattering. *Phys. Rev. Lett.* **124**, 013002 (2020).

67. S. Giri, J. C. Tremblay, G. Dixit, Imaging charge migration in chiral molecules using time-resolved x-ray diffraction. *Phys. Rev. A* **104**, 053115 (2021).

68. J. R. Rouxel, D. Keefer, S. Mukamel, Signatures of electronic and nuclear coherences in ultrafast molecular x-ray and electron diffraction. *Struct. Dyn.* **8**, 014101 (2021).

69. S. Giri, J. C. Tremblay, G. Dixit, Probing the effect of molecular structure saddling on ultrafast charge migration via time-resolved x-ray diffraction. *Phys. Rev. A* **106**, 033120 (2022).

70. J. C. Tremblay, A. Blanc, P. Krause, S. Giri, G. Dixit, Probing electronic symmetry reduction during charge migration via time-resolved x-ray diffraction. *ChemPhysChem* **24**, e202200463 (2023).

71. M. Centurion, T. J. A. Wolf, J. Yang, Ultrafast imaging of molecules with electron diffraction. *Annu. Rev. Phys. Chem.* **73**, 21–42 (2022).

72. M. T. Hassan, Lightwave electronics: Attosecond optical switching. *ACS Photonics* **11**, 334–338 (2024).

73. D. Hui, H. Alqattan, S. Zhang, V. Pervak, E. Chowdhury, M. T. Hassan, Ultrafast optical switching and data encoding on synthesized light *fields Sci. Adv.* **9**, eadf1015 (2023).

74. R. R. Nair, P. Blake, A. N. Grigorenko, K. S. Novoselov, T. J. Booth, T. Stauber, N. M. R. Peres, A. K. Geim, Fine structure constant defines visual transparency of graphene. *Science*. **320**, 1308–1308 (2008).
